# Increased Dead Space Ventilation as a Contributing Factor to Persistent Exercise Limitation in Patients with a Left Ventricular Assist Device

**DOI:** 10.3390/jcm12113658

**Published:** 2023-05-25

**Authors:** Simon Wernhart, Bastian Balcer, Tienush Rassaf, Peter Luedike

**Affiliations:** Department of Cardiology and Vascular Medicine, West German Heart and Vascular Center, University Hospital Essen, University Duisburg-Essen, Hufelandstrasse 55, 45147 Essen, Germany; bastian.balcer@uk-essen.de (B.B.); tienush.rassaf@uk-essen.de (T.R.); peter.luedike@uk-essen.de (P.L.)

**Keywords:** LVAD, HFrEF, V_D_/V_T_, RV–PA uncoupling

## Abstract

(1) Background: The exercise capacity of patients with a left ventricular assist device (LVAD) remains limited despite mechanical support. Higher dead space ventilation (V_D_/V_T_) may be a surrogate for right ventricular to pulmonary artery uncoupling (RV–PA) during cardiopulmonary exercise testing (CPET) to explain persistent exercise limitations. (2) Methods: We investigated 197 patients with heart failure and reduced ejection fraction with (*n* = 89) and without (HFrEF, *n* = 108) LVAD. As a primary outcome NTproBNP, CPET, and echocardiographic variables were analyzed for their potential to discriminate between HFrEF and LVAD. As a secondary outcome CPET variables were evaluated for a composite of hospitalization due to worsening heart failure and overall mortality over 22 months. (3) Results: NTproBNP (OR 0.6315, 0.5037–0.7647) and RV function (OR 0.45, 0.34–0.56) discriminated between LVAD and HFrEF. The rise of endtidal CO_2_ (OR 4.25, 1.31–15.81) and V_D_/V_T_ (OR 1.23, 1.10–1.40) were higher in LVAD patients. Group (OR 2.01, 1.07–3.85), VE/VCO_2_ (OR 1.04, 1.00–1.08), and ventilatory power (OR 0.74, 0.55–0.98) were best associated with rehospitalization and mortality. (4) Conclusions: LVAD patients displayed higher V_D_/V_T_ compared to HFrEF. Higher V_D_/V_T_ as a surrogate for RV–PA uncoupling could be another marker of persistent exercise limitations in LVAD patients.

## 1. Introduction

Although an improvement in mortality has been achieved through the implantation of left ventricular assist devices (LVAD) in patients with heart failure and reduced ejection fraction (HFrEF), exercise capacity (expressed by peak oxygen consumption, VO_2peak_) remains limited in this population [1,2]. Among others, this is caused by an inadequate increase in LVAD pump flow resulting in an insufficient increase in cardiac output [1] but also through hampered chronotropic competence [3]. Persistent alveolar hypoperfusion, which is demonstrated by the surrogate marker of an insufficient increase in endtidal CO_2_ (PETCO_2_) during cardiopulmonary exercise testing (CPET), has been shown in an LVAD population [4]. In addition to reduced VO_2peak_ [5], a blunted peak systolic blood pressure increase has been demonstrated in LVAD patients [6].

In HFrEF patients variables of CPET have been shown to have an impact on cardiovascular morbidity and mortality [7,8,9]. Compound variables, such as circulatory power (CP, peak systolic pressure x VO_2peak_) [9] and ventilatory power (VP, peak systolic pressure/VE/VCO_2_) [10] have been associated with mortality in HFrEF, but they have not been investigated in LVAD patients. As pathophysiology may differ between patients with and without left ventricular support, we aimed to investigate which echocardiographic, laboratory, and CPET variables were most suited to differentiate between HFrEF and LVAD patients and whether these variables were associated with rehospitalization and mortality in the groups. Although perfusion is increased by LVAD support, we hypothesized that impaired ventilatory mechanics, such as increased dead space ventilation, could be another contributing factor to explain persistent exercise intolerance in LVAD patients despite circulatory support.

## 2. Materials and Methods

### 2.1. Setting and Participants

We included patients above 18 years of age with reduced left ventricular ejection fraction (<40%) with (LVAD) and without (HFrEF) LVAD support over an observational period of 22 months. Patients of our in- and outpatient clinic undergoing elective CPET were included. To undergo CPET, patients had to be clinically euvolemic and free from systemic infection, which had to be clinically verified by a senior physician. Patients with EF ≥ 40% and younger age (<18 years) were excluded. The study protocol conforms to the ethical guidelines of the 1975 Declaration of Helsinki and was approved by the local ethics committee of the University Duisburg-Essen, Germany (22-10562-BO).

### 2.2. Cardiopulmonary Exercise Protocol

A ramp protocol on a bicycle ergometer (eBike II, GE Healthcare, Chicago, IL, USA) was performed with an estimated duration of 8–12 min, starting at a workload of 10 W with an increment of 10 W/min and a pedaling rate of 60 rounds per minute. Respiratory gas exchange was measured breath by breath using a metabolic cart interface (VyntusTM CPX Metabolic Cart, Vyaire Medical, Hoechberg, Germany). Ventilatory thresholds and data interpretations were performed by an exercise physiologist (SentrySuiteTM Software Solution, VyaireTM Medical). The percentage of age-predicted VO_2peak_ was calculated using the Wasserman–Hansen equation [11], the exercise oscillatory ventilation (EOV) was determined according to a previously described algorithm [12], the O_2_ pulse was related to body weight and multiplied by 100 for better readability [13], and plateauing of the O_2_ pulse was visually assessed by a flattening of the curve. The oxygen equivalent at the first ventilatory threshold (EqO_2_ at VT1) [14,15] and oxygen uptake efficiency slope (OUES), the relation of oxygen uptake, and the logarithmic minute ventilation [16] were assessed as previously recommended. We defined chronotropic incompetence (CI) as a lack of heart rate increase above 80% of the predicted heart rate during exertional exercise testing. A minimal increase in PETCO_2_ > 3 mmHg during exercise was expected for sufficient alveolar perfusion during exercise [14]. Dead space ventilation was estimated from endtidal CO_2_, capillary CO_2_ from the hyperemic ear as an approximation of arterial CO_2_ (p_a_CO_2_), tidal volume (V_T_), and dead space of the breathing valve (0.075 L).

V_D_/V_T_ = [(p_a_CO_2_ − PETCO_2_)/paCO_2_] − V_Bv_/(V_T_ − V_Bv_)

Exercise tests were performed until maximal exertion, defined as a respiratory exchange ratio (RER) > 1.05. Criteria for premature exercise termination were defined according to current guidelines [14]. Blood pressure was measured using a standard upper arm cuff. Patients were advised to take their morning medication on the day of exercise testing to simulate patients’ daily routine. Patients were advised to fast for at least three hours prior to CPET examination.

### 2.3. Co-Variable Assessment

Laboratory values and transthoracic echocardiography were performed within 48 h of CPET. Echocardiography was performed by an experienced non-invasive cardiologist according to established recommendations [17]. As image quality in LVAD patients can be challenging, the left ventricular ejection fraction (LVEF) was obtained using 2D-guided linear measurements in the parasternal long axis according to current guidelines [18]. The severity of relevant (at least grade 2) valve dysfunction was assessed qualitatively and semi-quantitatively according to current recommendations [19]. Tricuspid annular plane systolic excursion (TAPSE) was used as a surrogate for right ventricular function. 

We aimed to delineate laboratory, echocardiographic, and CPET markers to differentiate between HFrEF and LVAD. The potential of such variables to predict the combined outcome as a composite of hospitalization due to worsening heart failure and overall mortality during the observation period of 22 months was analyzed. We hypothesized that impaired ventilatory mechanics in the form of higher dead space ventilation would discriminate between HFrEF and LVAD patients. Higher dead space ventilation may serve as a non-invasive variable to detect right ventricular to pulmonary artery (RV–PA) uncoupling as a contributing factor to persistent exercise intolerance in LVAD patients.

### 2.4. Statistical Methods

SPSS (IBM Corp. Released 2016. IBM SPSS Statistics for Windows, Version 24.0. Armonk, NY, USA: IBM Corp.) and the R-program [20] were used for data analysis and graphical illustration. Baseline characteristics were assessed using descriptive statistics and the normal distribution was tested using the Shapiro–Wilk test. The effects of selected outcome variables on groups were evaluated using the Fisher exact test (nominal scale). A non-parametric U-test was applied to evaluate differences between groups in quantitative measurements (ratio scale). A level of significance α was set at 0.05. Multivariable logistic regression models were derived for variables showing significant differences between groups in univariate analysis and with suspected clinical relevance. Using the Akaike information criterion (AIC) multiple backwards eliminations were performed to exclude variables with minor impact to discriminate between groups. Receiver operating characteristics (ROCs) were calculated from the reduced model and the area under the curve (AUC) was determined. Through clinical prioritization selected variables were integrated into a final model and the contribution of individual variables to discriminate between groups was displayed using nomograms. A cut-off to differentiate overall mortality by VO_2peak_ between LVAD and HFrEF was chosen using the Youden criterion.

## 3. Results

### 3.1. Baseline Characteristics

**Figure 1:**
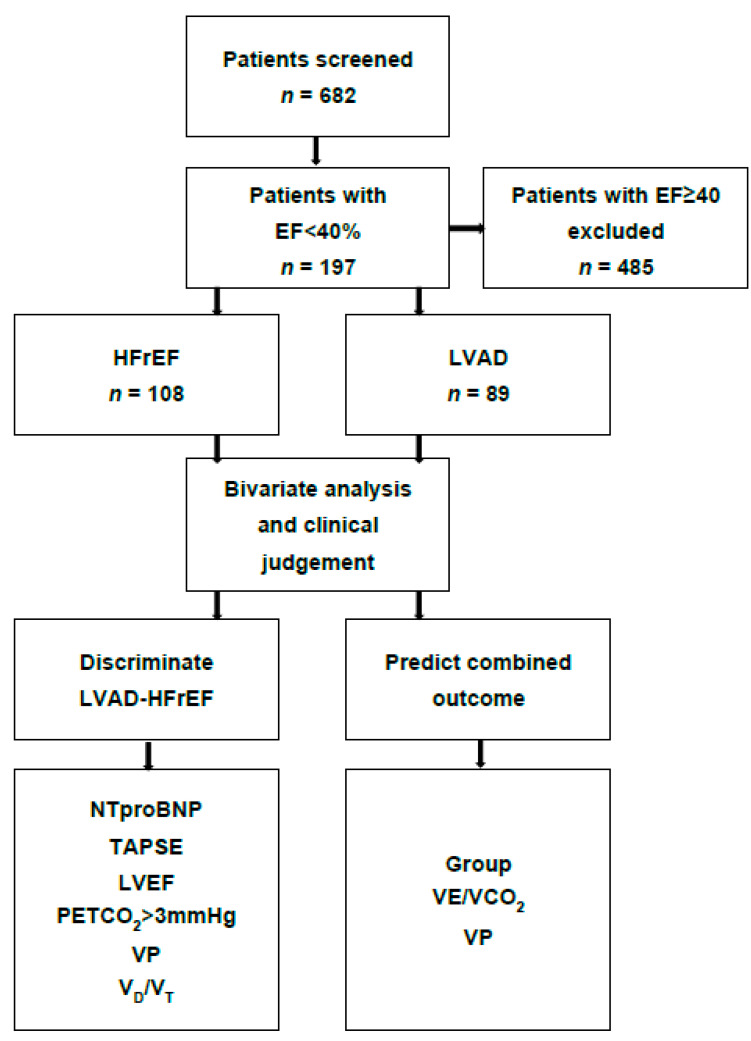
Flowchart of study inclusion. Combined outcome: composite of cardiovascular rehospitalization and mortality. EF: left ventricular ejection fraction. Group: patients with reduced left ventricular ejection fraction with and without a left ventricular assist device. HFrEF: heart failure with reduced ejection fraction (without left ventricular assist device support). LVAD: left ventricular assist device. NTproBNP: N-terminal prohormone of brain natriuretic peptide. PETCO_2_: endtidal carbon dioxide as a surrogate for alveolar perfusion. TAPSE: tricuspid annular plane systolic excursion. V_D_/V_T_: dead space ventilation during exercise. VE/VCO_2_: minute ventilation per carbon dioxide production. VP: ventilatory power (the ratio of peak systolic pressure and VE/VCO_2_).

A total of 197 patients (108 HFrEF and 89 LVAD) were included in the final analysis (Figure 1). 

The time since LVAD implantation was 25 ± 3.4 months. Atrial fibrillation (*p* < 0.001) and coronary artery disease (*p* = 0.031) were more prevalent in LVAD than HFrEF patients. Listing for heart transplantation was more prominent in LVAD patients (*p* = 0.037), while NTproBNP (*p* = 0.003), hemoglobin levels, left ventricular ejection fraction (LVEF), TAPSE, and the percentage of valve dysfunctions (all *p* < 0.001) were lower in this group (Table 1, interquartile ranges Table A1). 

TAPSE did not differ in LVAD and HFrEF patients depending on etiology of heart failure (ischemic vs. non-ischemic in LVAD *p* = 0.34 and in HFrEF *p* = 0.56). TAPSE was lower in LVAD (*p* = 0.03) and HFrEF (*p* = 0.02) patients with atrial fibrillation. Similarly, TAPSE was lower in the presence of valvular dysfunction in HFrEF (*p* = 0.02) and LVAD (*p* = 0.02) patients. Indications for LVAD implantation were destination therapy due to advanced heart failure in 69.7% (*n* = 62) and bridge-to-transplant in 30.3% (*n* = 27); none of the patients had been transplanted at the time of study termination. CRT-D had been implanted in 41.7% (*n* = 45) of HFrEF patients, while no CRT-P implantations had been performed. TAPSE was higher in patients with CRT-D compared to HFrEF patients without a device (*p* = 0.04). Rehospitalization due to worsening heart failure did not differ between LVAD (46.1%, *n* = 41) and HFrEF (39.8%, *n* = 43) patients (*p* = 0.39). There was no mortality difference between HFrEF (5.6%, *n* = 6) and LVAD (11.2%. *n* = 10, *p* = 0.19). Four patients in the HFrEF group died of cardiac shock due to ischemic events and two of cancer sequelae (one with acute respiratory decompensation and one due to tumor obstruction). In the LVAD group two patients died of an intracranial hemorrhage, six died of sepsis, and two died of the sequelae of gastrointestinal bleeding resulting in mixed cardiac and hemorrhagic shock.

### 3.2. Bivariate Analysis of CPET Parameters between Groups

Higher peak systolic pressure (*p* < 0.01), dead space ventilation (V_D_/V_T_, *p* < 0.01), and a higher percentage of PETCO_2_ increase >3 mmHg during exercise (*p* = 0.041) was found in LVAD patients. Peak performance (P_max_, *p* = 0.05) was higher in HFrEF, but VO_2peak_ (*p* = 0.11) did not differ among groups. The percentage of chronotropic incompetence (*p* = 0.76) and VE/VCO_2_ (*p* = 0.06) did not differ between groups (Table 2). VP was higher in LVAD patients (*p* < 0.01), while there was no difference in CP (*p* = 0.21, Table 2, for interquartile ranges see Table A2).

### 3.3. Discrimination between LVAD and HFrEF

Based on bivariate analysis and clinical judgement, we included NTproBNP, TAPSE, LVEF, PETCO_2_, VO_2peak_, VP, and V_D_/V_T_ into a logistic regression model. VO_2peak_, VP, and LVEF did not show discriminating power between the groups (Table A3), while NTproBNP, TAPSE, PETCO_2_, and V_D_/V_T_ differed well between the groups (Table 3).

**Figure 2:**
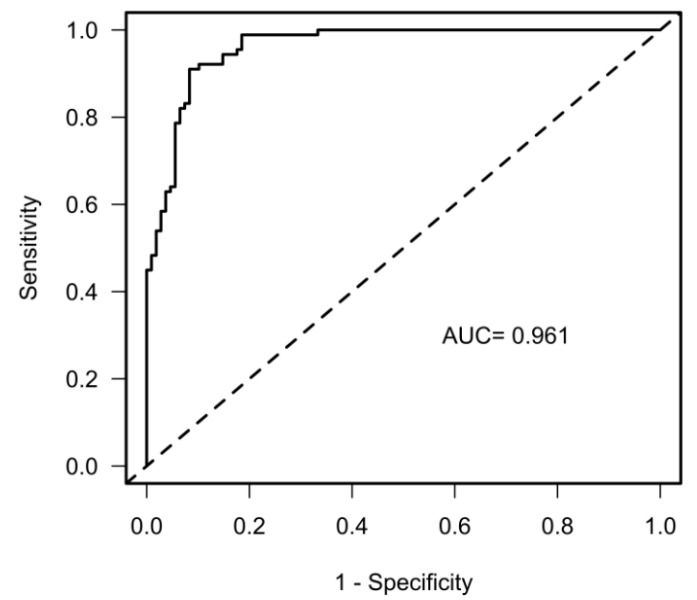
Discrimination between patients with (LVAD) and without (HFrEF) a left ventricular assist device with reduced left ventricular ejection fraction. Receiver operating characteristics showing the area under the curve (AUC).

The overall discriminating power of the multivariable model using the ROCs was AUC = 0.96 (CI 0.94–0.99 R2 = 0.79, Figure 2). 

**Figure 3:**
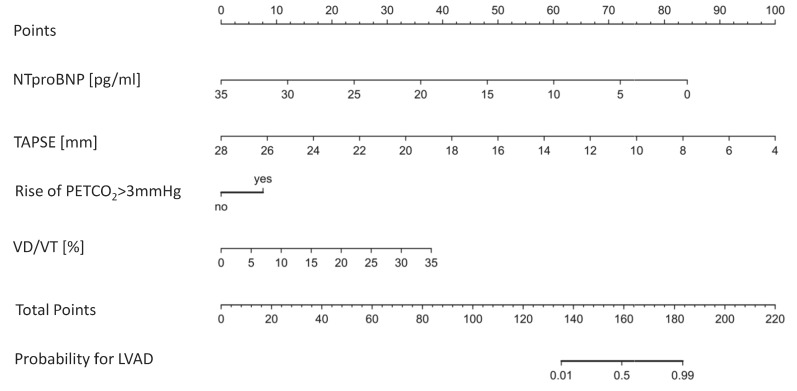
Discrimination between patients with (LVAD) and without (HFrEF) a left ventricular assist device with reduced left ventricular ejection fraction. Nomogram of variables to discriminate between groups. NTproBNP: N-terminal prohormone of brain natriuretic peptide. PETCO_2_: endtidal carbon dioxide as a surrogate for alveolar perfusion. TAPSE: tricuspid annular plane systolic excursion. V_D_/V_T_: dead space ventilation during exercise.

A nomogram to discriminate groups is illustrated in Figure 3 (for the full logistic regression model see Table A3).

### 3.4. CPET Variables to Predict the Combined Outcome

Based on prior analysis of CPET variables and clinical judgement we selected the factors group, VE/VCO_2_, VP, PETCO_2_, V_D_/V_T_, and VO_2peak_ as the gold standard for exercise capacity, for logistic regression analysis (Table A4). Only group, VE/VCO_2_, and VP showed an impact on the combined outcome (Table 4). 

**Figure 4:**
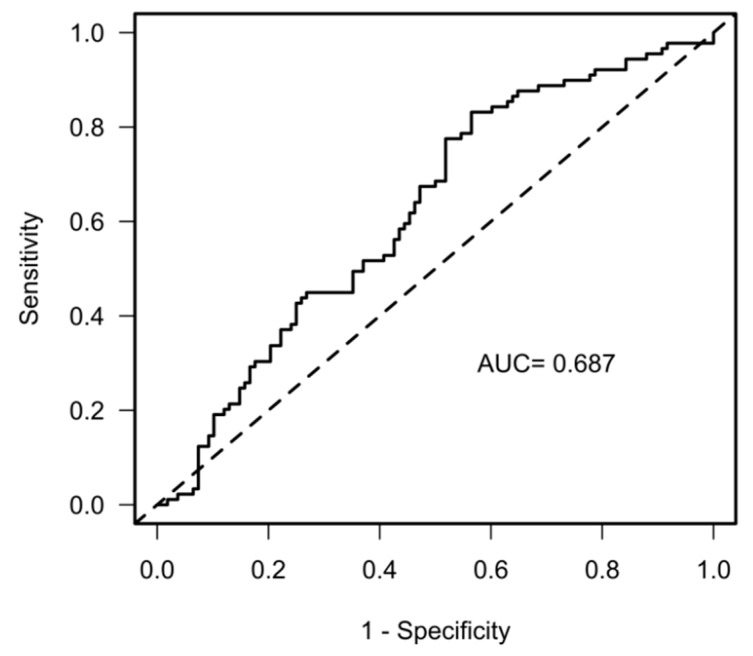
Prediction of the composite endpoint cardiovascular rehospitalization and mortality. Receiver operating characteristics showing the area under the curve (AUC).

The predictive power of the multivariable model using the ROCs was AUC = 0.69 (CI 0.62–0.77, R2 = 0.16, Figure 4). 

**Figure 5:**
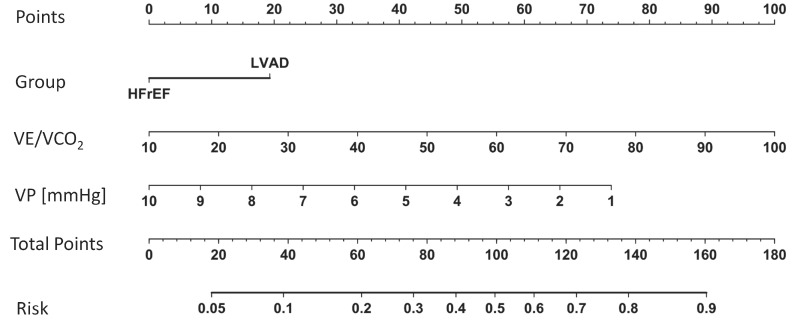
Nomogram of variables to predict the composite endpoint cardiovascular rehospitalization and mortality. HFrEF: patients with reduced left ventricular ejection fraction without an assist device. LVAD: patients with reduced left ventricular ejection fraction and a left ventricular assist device. VE/VCO_2_: minute ventilation per carbon dioxide production. VP: ventilatory power (the ratio of peak systolic pressure and VE/VCO_2_).

A nomogram to illustrate the predictive impact of the model is depicted in Figure 5 (for the full logistic regression model see Table A4).

**Figure 6:**
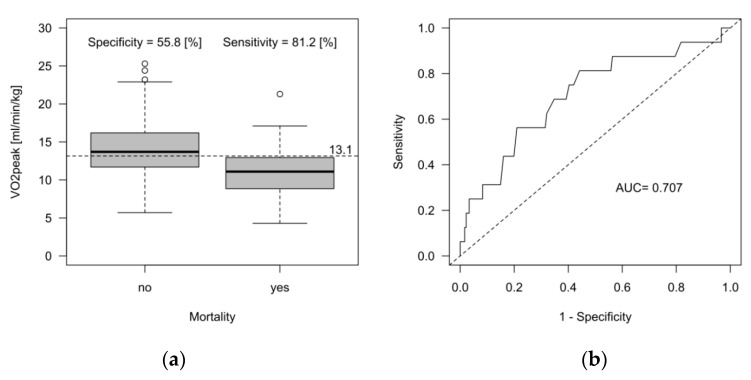
Peak oxygen consumption (VO_2peak_) to predict overall mortality. (**a**) Box plot to illustrate the relationship between VO_2peak_ and overall mortality in the entire population. The optimal cut-off, determined by the Youden criterion, is 13.1 mL/min/kg. (**b**) Receiver operating characteristics showing the area under the curve (AUC). The dashed horizontal line represents the calculated cut-off based on the study population.

CPET variables were also included in a regression modeling step for overall mortality (Table A5), with only VO_2peak_ showing a significant effect (*p* = 0.006, Figure 6a, Supplement 5). A VO_2peak_ of 13.1 mL/min/kg demonstrated a specificity of 55.8% and a sensitivity of 81.2% in the entire population of patients (AUC = 0.707, Figure 6b).

## 4. Discussion

Although mortality is improved by LVAD implantation in advanced heart failure, exercise limitations persist [1,2]. To implement adequate drug therapy, which may differ from the treatment of HFrEF, better knowledge of LVAD pathophysiology at rest and during exercise is necessary to delineate and potentially overcome persistent limitations despite circulatory support. We showed that LVAD patients display higher dead space ventilation despite increased alveolar perfusion during exercise. Together with lower TAPSE, as a surrogate for right ventricular function, higher dead space ventilation may serve as a non-invasive CPET variable to reveal RV–PA uncoupling as a contributing factor to persistent exercise intolerance in LVAD patients. We also show that VE/VCO_2_ and VP, both variables illustrating impaired ventilatory mechanics, were associated with hospitalization due to worsening heart failure and overall mortality.

### 4.1. Assessment of the Primary Outcome

We found that baseline (NTproBNP and TAPSE) and functional (V_D_/V_T_, PETCO_2_) variables can be useful to discriminate between HFrEF and LVAD patients. However, established variables such as LVEF and VO_2peak_ did not have such an effect. This is an important finding, since it has been shown that morbidity in HFrEF patients can be predicted by both VO_2peak_ and LVEF [21,22], but scarce data are available on PETCO_2_ and V_D_/V_T_ in LVAD patients. 

A small study comparing exercise performance between patients within two months after LVAD implantation (*n* = 26) and heart failure patients immediately after recompensation from acute heart failure (*n* = 30) found comparable VO_2peak_, but a trend towards lower OUES in LVAD patients suggesting higher ventilatory efforts in LVAD patients [23]. This is also supported by the observation that EOV, as a surrogate marker for elevated pulmonary artery wedge pressure and ventilatory inefficiency, does not seem to resolve following LVAD implantation [24]. Although we did not detect differences in EOV between the groups, we found that V_D_/V_T_ was higher in LVAD compared to HFrEF patients illustrating more inefficient ventilation during exercise. This occurred despite the finding that PETCO_2_, as a surrogate for alveolar perfusion, increased to a higher extent in the LVAD group. The latter may be explained by an increase in cardiac output achieved by the LVAD device. Higher V_D_/V_T_ was not the result of a higher prevalence of pulmonary disease in our LVAD patients, but this seems to be a consequence of wasted alveolar ventilation during exercise [4,25]. Compared to a recent study analyzing circulatory-ventilatory coupling in LVAD patients [4], our LVAD patients showed higher exercise capacity (VO_2peak_ 13.4 ± 3.5 mL/min/kg vs. 10.6 ± 1.7 mL/min/kg) and lower VE/VCO_2_ (36.7 ± 8.2 vs. 40.7 ± 5.2). VE/VCO_2_ has been shown to be a prognosticator of postoperative mortality and right ventricular dysfunction following LVAD implantation [26]. Although, higher V_D_/V_T_ can be the result of alveolar hypoperfusion due to reduced cardiac output during exercise; it can also be affected by alterations in preload, impaired contractility of the right ventricle, as well as exercise induced pulmonary hypertension [4]. V_D_/V_T_ may be suggested as a sensitive marker to detect early reduction in ventilatory efficiency, even before PETCO_2_ and VE/VCO_2_ deteriorate. Thus, V_D_/V_T_ should be implemented to risk stratify LVAD patients. 

Our precise multicomponent prediction model (AUC = 96.1%) also included TAPSE, rather than LVEF, to discriminate between LVAD and HFrEF patients. This supports the existing literature as it has been previously shown that reduced right ventricular function is associated with postoperative morbidity in LVAD patients [26]. Future prediction models of cardiovascular morbidity in LVAD patients should also implement measures of preload assessment, such as an echocardiographic collapse of the vena cava before starting exercise testing. In our study LVEF did not play a pivotal role in determining morbidity or overall mortality. In summary, reduced TAPSE and higher V_D_/V_T_ in LVAD patients may be the correlates of right ventricular–pulmonary artery uncoupling during exercise. Applying a nomogram for the risk to reach the composite outcome into clinical practice may be reasonable (Figure 5).

### 4.2. Assessment of the Composite Secondary Outcome

We also identified VE/VCO_2_ as a variable to predict rehospitalization and mortality in our population, which has been described previously [26]. However, our finding that VP may be a suitable (compound) variable to predict the outcome has not been investigated in an LVAD population. It has only been demonstrated that CP and VE/VCO_2_ were the best discriminators for the composite endpoint of transplantation, mechanical circulatory support (MCS) implantation, and death after one year in 400 HFrEF patients without an assist device (EF 29 ± 8%) [9]; VP was not investigated in this study. It has been shown that VP is associated with invasively measured mPAP (r = −0.427) in patients with suspected PH and that VP < 3.4 mmHg showed an OR of 4.5 for a mean pulmonary artery pressure ≥25 mmHg [27]. Furthermore, VP < 3.5 mmHg was shown to be associated with increased major cardiac events (AUC = 0.70) in heart failure patients with reduced ejection fraction [10]. Our population of HFrEF patients showed comparable VP (3.6 ± 1.3 mmHg) illustrating the importance of close follow-up to prevent cardiovascular events. However, our LVAD patients displayed higher values (4.4 ± 1.6 mmHg), which have to be interpreted with caution, since blood pressure was monitored using a regular upper arm cuff, which has limitations in LVAD patients due to pseudo-pulsatile blood flow. An assessment of VP in LVAD via simultaneous invasive arterial blood pressure monitoring is warranted.

### 4.3. Prediction of Overall Mortality

The major factor to evaluate overall mortality in our patients was VO_2peak_. Its predictive power has to be evaluated with care because the specificity was slightly above 50%. A retrospective multicenter study of 450 LVAD patients showed that a VO_2peak_ > 12 mL/min/kg and a VE/VCO_2_ slope > 35 were associated with a one-year survival of 100% [28]. Although VO_2peak_ was higher in our study (the optimal cut-off for mortality discrimination was 13.1 mL/min/kg), we observed device-specific complications during the 22 months follow-up. This may illustrate that other (exercise-independent) confounders play a role in assessing mortality. Mortality in LVAD was mainly driven by device-specific complications, such as sepsis or bleeding, which may not necessarily be associated with exercise capacity. Two deaths in the HFrEF group occurred due to cancer sequelae (one with acute respiratory decompensation and one due to tumor obstruction), which may not be associated with cardiac, circulatory, or ventilatory capacities during exercise. 

### 4.4. Clinical Implications of Group Differences between LVAD and HFrEF

We found TAPSE to be a major determinant of reduced exercise capacity in the groups irrespective of heart failure etiology. Lower TAPSE was associated with a higher rate of atrial fibrillation and valve dysfunction both in HFrEF and LVAD patients. Thus, guideline-directed treatment of valve dysfunction as well as adequate rate and/or rhythm control in HFrEF patients should be considered pivotal to delay the decline of exercise capacity and morbidity. Furthermore, CRT-D was associated with higher TAPSE in our HFrEF patients, which emphasizes the importance of adhering to heart failure guidelines to postpone right heart dysfunction and failure. Whether interventional treatment (e.g., edge-to-edge repair of atrioventricular valves) plays a role in improving performance and preventing rehospitalization in LVAD patients is not known. Inefficient ventilation (expressed by a VE/VCO_2_ increase) has been shown to be a determinant of rehospitalization in our study. Right ventricular dysfunction may exacerbate ventilation, but pulmonary co-morbidities may increase right ventricular afterload, which further deteriorates ventilatory efficiency. Furthermore, adequate RV–PA coupling is also facilitated by pulmonary and abdominal mechanics: the treatment of obstructive sleep apnea and obesity hypoventilation syndrome, which often co-exist with heart failure [22], need to be treated adequately to optimize dead space ventilation and diaphragmatic efficiency at rest and during exercise. In summary, the characterization of LVAD patients and their exercise performance properties is essential to facilitate and tailor medical as well as potential future device therapy in this vulnerable cohort. 

### 4.5. Limitations

There are limitations in this study: (1) We performed a retrospective data analysis, which was not powered for the primary outcome; (2) The analysis of compound variables CP and VP need to be interpreted with caution, since peak systolic pressure was not measured invasively but with a standard blood pressure cuff. Although these variables are promising, prospective studies comparing invasive and non-invasive blood pressure measurements are warranted; (3) Capillary blood gas analysis was not available to precisely detect diffusion limitations in the population. Thus, dead space ventilation was estimated from endtidal CO_2_; (4) Echocardiographic measurement of systolic pulmonary artery pressure to calculate TAPSE/sPAP could not be adequately achieved in LVAD patients due to limited imaging quality.

## 5. Conclusions

Compared to HFrEF, LVAD patients displayed reduced TAPSE and higher V_D_/V_T_ during exercise despite increased alveolar perfusion. Thus, RV–PA uncoupling as the correlate of reduced exercise capacity in LVAD patients could be driven by impaired ventilatory mechanics as an independent factor but also as the result of reduced right ventricular function. Ventilatory mechanics and their independent prognostic value in LVAD patients need to be further investigated.

## Figures and Tables

**Table 1:** Patient characteristics in the heart failure groups.

Medical History	HFrEF	LVAD	*p*-Value
	(*n* = 108)	(*n* = 89)	
Age [years]	51.7 ± 10.9	53.6 ± 10.1	*p* = 0.18
BMI [kg/m^2^]	28.0 ± 5.0	28.6 ± 4.3	*p* = 0.49
Women [%]	15.7 (*n* = 17)	14.6 (*n* = 13)	*p* = 0.85
Diabetes [%]	34.3 (*n* = 37)	31.5% (*n* = 28)	*p* = 0.76
Hypertension [%]	44.4 (*n* = 48)	46.1% (*n* = 41)	*p* = 0.89
AF [%]	23.1 (*n* = 25)	48.3 (*n* = 43)	*p* < 0.01 *
Smoking [%]	54.6 (*n* = 59)	57.3% (*n* = 51)	*p* = 0.77
CAD [%]	50.9 (*n* = 55)	66.3 (*n* = 59)	*p* = 0.03 *
NYHA class [%]			*p* = 0.55
I	0.9 (*n* = 1)	0.0 (*n* = 0)	
II	38.0 (*n* = 41)	36.0 (*n* = 32)	
III	57.4 (*n* = 62)	62.9 (*n* = 56)	
IV	3.7 (*n* = 4)	1.1 (*n* = 1)	
Listed for heart transplant	29.6 (*n* = 32)	44.9% (*n* = 40)	*p* = 0.04 *
Rehospitalization [%]	39.8% (*n* = 43)	46.1% (*n* = 41)	*p* = 0.39
Overall Mortality [%]	5.6% (*n* = 6)	11.2% (*n* = 10)	*p* = 0.19
BB, % patients (*n*)	95.4 (*n* = 103)	96.6 (*n* = 86)	*p* = 0.73
MRA [%]	87.0 (*n* = 94)	89.9 (*n* = 80)	*p* = 0.66
ACEi/ARB/ARNI [%]	95.4 (*n* = 103)	93.3 (*n* = 83)	*p* = 0.55
Loop diuretics [%]	78.7 (*n* = 85)	71.9 (*n* = 64)	*p* = 0.32
SGLT2 inhibitor	79.6 (*n* = 86)	49.4 (*n* = 44)	*p* < 0.01 *
Laboratory values			
NTproBNP [pg/mL]	3872.2 ± 5322.4	1889.1 ± 2408.2	*p* < 0.01 *
Hemoglobin [g/dl]	14.2 ± 2.5	12.9 ± 2.2	*p* < 0.01 *
eGFR [ml/min]	59.4 ± 14.8	59.2 ± 14.0	*p* = 0.44
Thrombocytes [/nl]	249.8 ± 274.9	235.2 ± 75.5	*p* = 0.41
Echocardiographic variables			
LVEF [%]	24.8 ± 7.8	20.7 ± 7.1	*p* < 0.01 *
TAPSE [mm]	17.4 ± 4.0	10.9 ± 2.3	*p* < 0.01 *
Valve dysfunction [%]	48.1 (*n* = 52)	12.4 (*n* = 11)	*p* < 0.01 *

Heart failure with reduced ejection fraction with (LVAD) and without a left ventricular assist device (HFrEF). AF: atrial fibrillation. CAD: coronary artery disease. BB: % of patients on beta-blockers. MRA: mineralocorticoid receptor antagonist. ACEi: angiotensin-converting enzyme inhibitor. ARB: angiotensin receptor blocker. ARNI: angiotensin receptor neprilysin inhibitor. eGFR: estimated glomerular filtration rate. LVEF: left ventricular ejection fraction. NTproBNP: N-terminal prohormone of brain natriuretic peptide. NYHA: New York Heart Failure Association class. SGLT2-inhibitor: sodium glucose co-transporter 2 inhibitor. TAPSE: tricuspid annular plane systolic excursion. Valve dysfunction: valve dysfunction was defined as the presence of ≥ grade II valve stenosis or insufficiency. Significance is denoted with an asterisk at alpha < 0.05. Differences in baseline and CPET characteristics were calculated using the Fisher exact and Mann–Whitney U-tests.

**Table 2:** Performance in cardiopulmonary exercise testing in the heart failure groups.

CPET Variables	HFrEF	LVAD	*p*-Value
	(*n* = 108)	(*n* = 89)	
CI [%]	31.4 (*n* = 34)	27.0 (*n* = 24)	*p* = 0.69
HR_max_ [beats/min]	117.5 ± 22.4	113.7 ± 25.2	*p* = 0.57
RR_sysmax_ [mmHg]	134.6 ± 35.3	155.7 ± 44.7	*p* < 0.01 *
RER	1.5 ± 4.3	1.1 ± 0.1	*p* = 0.70
VO_2peak_ [mL/min/kg]	14.3 ± 4.1	13.4 ± 3.5	*p* = 0.11
% of VO_2pred_	51.9 ± 14.9	49.7 ± 13.0	*p* = 0.33
% of pred VO_2_ at VT1	34.2 ± 8.9	33.7 ± 8.7	*p* = 0.68
P_max_ [W]	97.2 ± 40.9	84.2 ± 31.9	*p* = 0.05 *
VE [l]	60.9 ± 18.5	54.4 ± 16.1	*p* = 0.02 *
VE/VCO_2_	40.9 ± 12.9	36.7 ± 8.2	*p* = 0.06
VO_2_/W [mL/min/W]	8.8 ± 3.3	8.5 ± 2.8	*p* = 0.57
Plateau of O_2_ pulse [%]	61.1 (*n* = 66)	70.8 (*n* = 63)	*p* = 0.18
O_2_ pulsemax [mL/beat/kg × 100]	11.1 ± 3.7	10.7 ± 3.3	*p* = 0.36
EqO_2_ at VT1	27.1 ± 5.8	26.2 ± 5.2	*p* = 0.29
OUES	1.5 ± 0.6	1.4 ± 0.5	*p* = 0.19
V_D_/V_T_ [%]	14.2 ± 5.7	16.0 ± 3.9	*p* < 0.01 *
BR FEV_1_ [%]	37.3 ± 16.6	35.6 ± 18.9	*p* = 0.74
Circulatory power [mL/kg/min × mmHg]	1933.7 ± 729.4	2073.2 ± 774.3	*p* = 0.21
Ventilatory power [mmHg]	3.6 ± 1.3	4.4 ± 1.6	*p* < 0.01 *
EOV [%]	51.9 (*n* = 56)	51.7 (*n* = 46)	*p* = 0.99
PETCO_2_ > 3 mmHg [%]	53.7 (*n* = 58)	68.5 (*n* = 61)	*p* = 0.04 *

Heart failure with reduced ejection fraction with (LVAD) and without a left ventricular assist device (HFrEF). BR FEV_1_: breathing reserve based on resting forced expiratory volume in one second. CI: chronotropic incompetence. Circulatory power: peak oxygen consumption × peak systolic blood pressure. CPET: cardiopulmonary exercise testing. HR_max_: maximal heart rate at peak exercise. RR_sysmax_: systolic pressure at peak exercise. VO_2peak_: peak oxygen consumption. % of VO_2pred_: % of predicted VO_2peak_. P_max_: peak performance. O_2_ pulsemax: O_2_ pulse at peak exercise related to body weight. EqO_2_ at VT1: oxygen equivalent at the first ventilatory threshold. OUES: oxygen uptake efficiency slope. EOV: exercise oscillatory ventilation. % of pred VO_2_ at VT1: percent of predicted oxygen uptake at the first ventilatory threshold. Plateau of O_2_ pulse: flattening of the O_2_ pulse curve during exercise. VE: respiratory minute volume. PETCO_2_: endtidal carbon dioxide. RER: respiratory exchange ratio. Ventilatory power: peak systolic pressure/VE/VCO_2_. V_D_/V_T_: dead space ventilation during exercise. Mean values are depicted with standard deviations in round brackets. Significance is denoted with an asterisk at alpha < 0.05. Differences in baseline and CPET characteristics were calculated using the Fisher exact and Mann–Whitney U-tests.

**Table 3:** Reduced logistic regression model to discriminate LVAD and HFrEF.

Variable	Odds Ratio	95% Confidence Limits
NTproBNP [pg/mL]	0.63 *	0.50–0.77
TAPSE [mm]	0.45 *	0.34–0.56
PETCO_2_ > 3 mmHg	4.25 *	1.31–15.81
V_D_/V_T_ [%]	1.23 *	1.10–1.40

Discrimination between patients with reduced left ventricular ejection fraction with a left ventricular assist device (LVAD) or without an assist device (HFrEF). NTproBNP: N-terminal prohormone of brain natriuretic peptide. PETCO_2_: endtidal carbon dioxide. TAPSE: tricuspid annular plane systolic excursion. V_D_/V_T_: dead space ventilation during exercise. Odds ratios and confidence intervals are depicted. Significance is denoted with an asterisk.

**Table 4:** Reduced logistic regression model to predict the combined outcome.

Variable	Odds Ratio	95% Confidence Limits
Group LVAD	2.01 *	1.07–3.85
VE/VCO_2_	1.04 *	1.00–1.08
VP [mmHg]	0.74 *	0.55–0.98

Variables to predict the combined outcome cardiovascular rehospitalization and overall mortality across 22 months of follow-up. LVAD: left ventricular assist device. VE/VCO_2_: ratio of minute ventilation and carbon dioxide production. VP: ventilatory power, as the product of peak systolic pressure and VE/VCO_2_. Odds ratios and confidence intervals are depicted. Significance is denoted with an asterisk.

## Data Availability

Data will be made available by the corresponding author upon reasonable request.

## Appendix A

**Table A1:** Interquartile ranges of baseline variables between groups.

Baseline Variables	Min	Q25	Q50	Q75	Max
Age [years]					
HFrEF (*n* = 108)	24.0	43.0	53.0	60.0	80.0
LVAD (*n* = 89)	22.0	49.0	54.0	62.0	71.0
BMI [kg/m^2^]					
HFrEF (*n* = 108)	15.0	25.0	29.0	31.0	40.0
LVAD (*n* = 89)	19.0	25.0	29.0	31.0	40.0
NTproBNP [pg/mL]					
HFrEF (*n* = 108)	109.0	657.0	1839.0	4894.3	34,781.0
LVAD (*n* = 89)	35.0	539.5	937.0	2030.0	10,702.0
Hb [g/dL]					
HFrEF (*n* = 108)	8.5	12.5	14.6	15.7	19.7
LVAD (*n* = 89)	7.6	11.8	12.8	14.7	17.2
Thrombocytes [/nL]					
HFrEF (*n* = 108)	62.0	175.0	218.5	257.5	983.0
LVAD (*n* = 89)	111.0	183.0	220.0	277.5	485.0
GFR [mL/min]					
HFrEF (*n* = 108)	23.0	51.0	65.0	72.0	78.0
LVAD (*n* = 89)	26.0	47.5	60.0	71.0	76.0
Echocardiographic variables	Min	Q25	Q50	Q75	Max
LVEF [%]					
HFrEF (*n* = 108)	10.0	20.0	25.0	30.0	33.0
LVAD (*n* = 89)	12.0	15.0	19.0	23.0	39.0
TAPSE [mm]					
HFrEF (*n* = 108)	6.0	15.0	18.0	20.8	28.0
LVAD (*n* = 89)	5.0	9.0	11.0	12.5	17.0

Patients with reduced left ventricular ejection fraction with a left ventricular assist device (LVAD) or without an assist device (HFrEF). BMI: body mass index. GFR: glomerular filtration rate. Hb: hemoglobin. HFrEF: heart failure with reduced ejection fraction. LVEF: left ventricular ejection fraction. NTproBNP: N-terminal prohormone of brain natriuretic peptide. TAPSE: tricuspid annular plane systolic excursion.

**Table A2:** Interquartile ranges of CPET variables between groups.

CPET Variables	Min	Q25	Q50	Q75	Max
P_max_ [W]					
HFrEF (*n* = 108)	25.0	67.3	94.0	127.8	202.0
LVAD (*n* = 89)	20.0	61.5	83.0	102.0	161.0
VE [L/min]					
HFrEF (*n* = 108)	25.0	47.0	61.0	73.0	107.0
LVAD (*n* = 89)	20.0	42.5	53.0	65.5	99.0
VO_2peak_ [mL/min/kg]					
HFrEF (*n* = 108)	5.7	11.5	14.0	16.8	25.3
LVAD (*n* = 89)	4.3	11.1	13.1	15.0	21.8
% of VO2predicted					
HFrEF (*n* = 108)	17.9	40.2	52.4	63.9	86.4
LVAD (*n* = 89)	20.5	39.8	50.5	58.9	86.1
% of VO2 at VT1					
HFrEF (*n* = 108)	10.0	28.0	34.0	40.0	56.0
LVAD (*n* = 89)	12.0	28.5	33.0	38.0	68.0
HRmax [beats/min]					
HFrEF (*n* = 108)	71.0	100.0	115.5	131.0	176.0
LVAD (*n* = 89)	36.0	101.0	117.0	129.0	171.0
RRsysmax [mmHg]					
HFrEF (*n* = 108)	74.0	110.0	130.0	151.8	260.0
LVAD (*n* = 89)	76.0	120.0	150.0	184.5	274.0
O_2_ pulsemax [mL/beat/kg × 100]					
HFrEF (*n* = 108)	3.9	8.5	10.9	13.6	21.6
LVAD (*n* = 89)	3.5	8.6	10.4	12.8	21.5
OUES					
HFrEF (*n* = 108)	0.3	1.0	1.4	1.9	3.1
LVAD (*n* = 89)	0.3	1.0	1.3	1.6	2.8
VE/VCO_2_					
HFrEF (*n* = 108)	23.0	32.1	38.0	45.3	94.2
LVAD (*n* = 89)	13.2	30.9	35.6	41.6	62.5
EqO_2_ at VT1					
HFrEF (*n* = 108)	18.0	23.1	25.7	30.3	52.0
LVAD (*n* = 89)	18.0	22.9	24.7	29.5	46.0
Circulatory power					
[ml/kg/min × mmHg]					
HFrEF (*n* = 108)	581.4	1329.9	1927.0	2452.2	4222.4
LVAD (*n* = 89)	567.6	1447.6	2034.9	2580.4	4438.8
Ventilatory power [mmHg]					
HFrEF (*n* = 108)	1.2	2.7	3.4	4.4	6.5
LVAD (*n* = 89)	1.8	3.2	4.6	5.2	9.5
Breathing reserve [%]					
HFrEF (*n* = 108)	0.0	26.0	36.5	50.0	75.0
LVAD (*n* = 89)	0.0	24.5	38.0	48.5	74.0
V_D_/V_T_ [%]					
HFrEF (*n* = 108)	1.0	10.3	14.0	17.0	31.0
LVAD (*n* = 89)	0.0	14.0	16.0	18.0	25.0

Circulatory power: product of peak oxygen consumption and peak systolic blood pressure. HR_max_: peak heart rate. ΔO_2_ pulsemax: change in O_2_ pulse at peak exercise related to body weight. P_max_: peak performance. Peak oxygen pulse. EqO_2_ at VT1: oxygen equivalent at the first ventilatory threshold. HFrEF: heart failure with reduced ejection fraction without a left ventricular assist device. LVAD: left ventricular assist device. OUES: oxygen uptake efficiency slope. RR_sysmax_: peak systolic blood pressure. V_D_/V_T_: dead space ventilation during peak exercise. VE: minute ventilation. Ventilatory power: ratio of peak systolic blood pressure and VE/VCO_2_. VE/VCO_2_: minute ventilation per carbon dioxide production. VO_2peak_: peak oxygen consumption. VT1: first ventilatory threshold.

**Table A3:** Full logistic regression model to discriminate LVAD and HFrEF.

LVAD vs. HFrEF	Estimate	Standard Error	*p*-Value
Intercept	6.97	2.47	*p* < 0.01 *
VO_2peak_ [mL/min/kg]	−0.04	0.09	*p* = 0.66
NTproBNP [pg/mL]	−0.01	0.01	*p* < 0.01 *
TAPSE [mm]	−0.79	0.13	*p* < 0.01 *
LVEF [%]	−0.01	0.04	*p* = 0.83
PETCO_2_ > 3 mmHg	1.30	0.65	*p* = 0.05 *
VP [mmHg]	0.38	0.23	*p* = 0.11
V_D_/V_T_ [%]	0.23	0.07	*p* < 0.01 *

Discrimination between patients with reduced left ventricular ejection fraction with a left ventricular assist device (LVAD) or without an assist device (HFrEF). LVEF: left ventricular assist device. NTproBNP: N-terminal prohormone of brain natriuretic peptide. PETCO_2_: endtidal carbon dioxide. TAPSE: tricuspid annular plane systolic excursion. V_D_/V_T_: dead space ventilation during exercise. VO_2peak_: peak oxygen consumption. VP: ventilatory power, as the product of peak systolic pressure and VE/VCO_2_. Significance is denoted with an asterisk.

**Table A4:** Full logistic regression model to predict the combined outcome.

	Estimate	Standard Error	*p*-Value
Intercept	0.31	1.69	*p* = 0.86
Group-LVAD	0.69	0.35	*p* = 0.05 *
VO_2peak_ [mL/min/kg]	−0.07	0.05	*p* = 0.23
VE/VCO_2_	0.04	0.02	*p* = 0.07
VP [mmHg]	−0.30	0.15	*p* = 0.04 *
PETCO_2_ > 3 mmHg	0.23	0.35	*p* = 0.52
V_D_/V_T_ [%]	−0.04	0.04	*p* = 0.34

Full logistic regression model to predict the combined outcome of cardiovascular rehospitalization and overall mortality across 22 months of follow-up. LVAD: left ventricular assist device. PETCO_2_: endtidal carbon dioxide. V_D_/V_T_: dead space ventilation during exercise. VE/VCO_2_: ratio of minute ventilation and carbon dioxide production. VO_2peak_: peak oxygen consumption. VP: ventilatory power, as the product of peak systolic pressure and VE/VCO_2_. Odds ratios and confidence intervals are depicted. Significance is denoted with an asterisk.

**Table A5:** Full logistic regression model to predict overall mortality.

	Estimate	Standard Error	*p*-Value
Intercept	0.31	1.69	*p* = 0.86
Group-LVAD	0.69	0.35	*p* = 0.05 *
VO_2peak_ [mL/min/kg]	−0.07	0.05	*p* = 0.23
VE/VCO_2_	0.04	0.02	*p* = 0.07
VP [mmHg]	−0.30	0.15	*p* = 0.04 *
PETCO_2_ > 3 mmHg	0.23	0.35	*p* = 0.52
V_D_/V_T_ [%]	−0.04	0.04	*p* = 0.34

Full logistic regression model to predict the overall mortality across 22 months of follow-up. LVAD: left ventricular assist device. PETCO_2_: endtidal carbon dioxide. V_D_/V_T_: dead space ventilation during exercise. VE/VCO_2_: ratio of minute ventilation and carbon dioxide production. VO_2peak_: peak oxygen consumption. VP: ventilatory power, as the product of peak systolic pressure and VE/VCO_2_. Significance is denoted with an asterisk.
